# Signaling Pathways Used by the Specialized Pro-Resolving Mediator Maresin 2 Regulate Goblet Cell Function: Comparison with Maresin 1

**DOI:** 10.3390/ijms23116233

**Published:** 2022-06-02

**Authors:** Markus V. Olsen, Anne V. Lyngstadaas, Jeffrey A. Bair, Robin R. Hodges, Tor P. Utheim, Charles N. Serhan, Darlene A. Dartt

**Affiliations:** 1 Schepens Eye Research Institute/Massachusetts Eye and Ear, Department of Ophthalmology, Harvard Medical School, Boston, MA 02114, USA; marvice77@hotmail.com (M.V.O.); viktoria.lyngstadaas@gmail.com (A.V.L.); jeffrey_bair@meei.harvard.edu (J.A.B.); robin_hodges@meei.harvard.edu (R.R.H.); t.p.utheim@odont.uio.no (T.P.U.); 2 Institute of Clinical Medicine, Faculty of Medicine, University of Oslo, 0316 Oslo, Norway; 3 Department of Medical Biochemistry, Oslo University Hospital, 0424 Oslo, Norway; 4 Department of Plastic and Reconstructive Surgery, Oslo University Hospital, 0424 Oslo, Norway; 5 Center for Experimental Therapeutics and Reperfusion Injury, Department of Anesthesia, Brigham and Women’s Hospital and Harvard Medical School, Boston, MA 02115, USA; cserhan@bwh.harvard.edu; 6 Department of Oral Biology, Faculty of Dentistry, University of Oslo, 0316 Oslo, Norway

**Keywords:** mucin secretion, intracellular Ca^2+^, inflammation, epithelial cell, tear film, eye

## Abstract

Specialized pro-resolving mediators (SPMs), including Maresins (MaR)-1 and 2, contribute to tear film homeostasis and resolve conjunctival inflammation. We investigated MaR2′s signaling pathways in goblet cells (GC) from rat conjunctiva. Agonist-induced [Ca^2+^]_i_ and high-molecular weight glycoconjugate secretion were measured. MaR2 increased [Ca^2+^]_i_ and stimulated secretion. MaR2 and MaR1 stimulate conjunctival goblet cell function, especially secretion, by activating different but overlapping GPCR and signaling pathways, and furthermore counter-regulate histamine stimulated increase in [Ca^2+^]_i_. Thus, MaR2 and MaR1 play a role in maintaining the ocular surface and tear film homeostasis in health and disease. As MaR2 and MaR1 modulate conjunctival goblet cell function, they each may have potential as novel, but differing, options for the treatment of ocular surface inflammatory diseases including allergic conjunctivitis and dry eye disease. We conclude that in conjunctival GC MaR2 and MaR1, both increase the [Ca^2+^]_i_ and stimulate secretion to maintain homeostasis by using one set of different, but overlapping, signaling pathways to increase [Ca^2+^]_i_ and another set to stimulate secretion. MaR2 also resolves ocular allergy.

## 1. Introduction

The ocular surface including the cornea and conjunctiva is covered by a protective tear film. The innermost layer of the tear film is the mucous layer, which consists of electrolytes, water, and mucins produced by conjunctival goblet cells [1]. The mucins provide a critical line of defense from the external environment and play a role in maintaining health [2]. A disturbance in the homeostasis of mucin secretion has been described in a variety of inflammatory ocular surface diseases, including allergic conjunctivitis, Sjogren’s syndrome, and dry eye disease [2,3,4,5,6,7]. Disruption to mucin amount, structure, or hydration is deleterious to corneal clarity and hence vision. The resolution of inflammation is an active process with the production of pro-resolution mediators [8]. A group of lipid mediators called specialized pro-resolving mediators (SPMs), including the Maresins (MaRs), maintain homeostasis and counter regulate pro-inflammatory mediators in disease [9,10]. 

MaR1 and MaR2 are biosynthesized in macrophages and other tissues and are derived from the endogenous ω-3 fatty acid docosahexaenoic acid (DHA) [11]. MaR1 and MaR2 are synthesized through multiple enzymatic steps. Biosynthesis is initiated by 12-lipoxygenase (12-LOX), which converts DHA to 14-hydroperoxydocosahexaenoic acid. The two MaRs share a biosynthesis pathway until 13S, 14S-epoxy-maresin. 13S, 14S-epoxy-maresin is enzymatically converted to MaR1 by a hydrolase and to MaR2 by a soluble epoxide hydrolase [11,12]. Both MaRs consist of a carbon chain which is 22 carbons long, a carboxyl group, two hydroxyl groups, and six double bonds; however, the placement of the hydroxyl groups and double bonds are dissimilar. 

MaR1 and MaR2 function by limiting polymorphonuclear (PMN) infiltration and stimulating macrophage phagocytosis. By reducing the number of PMNs and removing apoptotic and necrotic cells the MaRs act to resolve inflammation [11,12]. In addition to pro-resolving effects, MaR1 stimulates regeneration and reduces pain. After surgical decapitation of planaria, MaR1 is biosynthesized which accelerates regeneration [13]. MaR1 reduces inflammatory and neuropathic pain by inhibition of TRPV1 [13]. Furthermore, MaR1 is present in human lymphoid tissue (spleen and lymph nodes) and human serum, indicating a possible role in the immune system [14]. Recent investigation of the actions of MaR1 on rat conjunctival goblet cells demonstrated that MaR1 increases [Ca^2+^]_i_ and stimulates glycoprotein secretion. MaR1 increased [Ca^2+^]_i_ and stimulated glycoprotein secretion by activating PLC and its downstream effectors, IP_3_, PKC, and by activation of PLD, Ca^2+^-calmodulin kinase (CaMK) II and extracellular regulated kinase (ERK) 1/2 [15]. 

In the present study, we investigated the action of MaR2 on cultured rat conjunctival goblet cells. To activate goblet cells and stimulate mucin secretion, one of the main signals is an increase in the intracellular Ca^2+^ concentration ([Ca^2+^]_i_) that triggers mucin secretion. Therefore, measuring the [Ca^2+^]_i_ was used as functional readout in the study presented in this manuscript. We identified the intracellular pathways MaR2 uses by measuring [Ca^2+^]_i_ and high molecular weight glycoprotein secretion including mucin secretion. We used pharmacologic inhibitors of different signaling pathways followed by addition of MaR2. Furthermore, the effect of MaR2 on histamine was investigated, because the MaRs are thought to play a central role in allergy [15]. MaR1 was used as a control for comparison. 

## 2. Results

### 2.1. Maresin 2 Increases Secretion in Rat Conjunctival Goblet Cells

**Figure 1:**
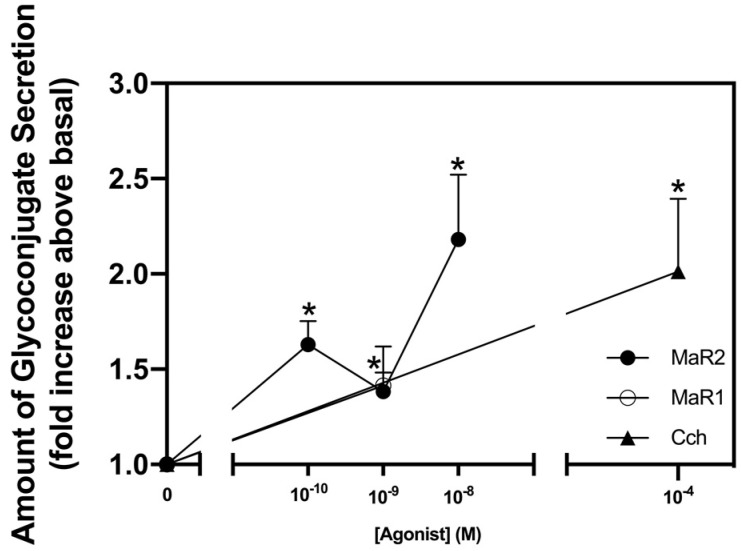
Maresin 2 (MaR2) stimulates glycoconjugate secretion. Rat conjunctival goblet cells were stimulated with either MaR2 (10^−10^–10^−8^ M) Maresin 1 (MaR1, 10^−8^ M), or carbachol (Cch, 10^−4^ M) for 2 hr. High molecular weight glycoprotein secretion was measured. Data are mean ± SEM from six experiments. * shows significance above basal. SEM, standard error of the mean.

The SPMs, lipoxin (LX)A_4_, resolvin (Rv)D1, RvE1 and MaR1 stimulate secretion from rat conjunctival goblet cells [15,16,17,18,19]. We compared the action of MaR2 (10^−10^ M–10^−8^ M) with that of the positive controls the cholinergic agonist carbachol (Cch) at 10^−4^ M and MaR1 at 10^−8^ M that are known to increase rat goblet cell secretion [15,20]. Cultured rat goblet cells were stimulated for 2 hr with MaR2, MaR1 or Cch. MaR2 significantly increased glycoconjugate secretion at 10^−10^ M (1.62 ± 0.12; *p* = 0.0005) and 10^−8^ M (2.18 ± 0.34; *p* = 0.006), but not at 10^−9^ M (1.38 ± 0.24; *p* = 0.14) (Figure 1; *n* = 6)). MaR1 and Cch each stimulated secretion. 

### 2.2. Maresin 2 Increases [Ca^2+^]_i_ That Stimulates Secretion in Rat Conjunctival Goblet Cells

**Figure 2:**
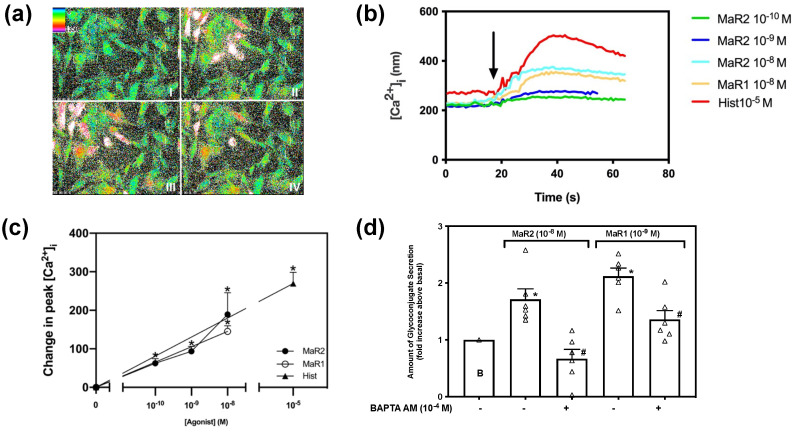
Maresin 2 increases [Ca^2+^]_i_ in rat conjunctival goblet cells. Pseudocolor images of rat conjunctival goblet cells stimulated with MaR2 (10^−8^ M) are shown in (**a**). MaR1 (10^−8^ M) is shown at four different times of stimulation. Panel I shows rat conjunctival goblet cell baseline [Ca^2+^]_i_ level before stimulation (AI), panel II 30 s after stimulation with MaR2 (10^−8^ M) (AII); panel III 50 s after stimulation (AIII); and panel IV 80 s after stimulation (AIV). Changes in [Ca^2+^]_i_ over time at different concentrations of MaR2 (10^−8^ M) are shown in (**b**). Changes in peak [Ca^2+^]_i_ after stimulation with MaR2 (10^−8^ M) are shown in (**c**) Goblet cells were preincubated with BAPTA/AM (10^−4^ M) and stimulated with MaR2 (10^−8^ M) or MaR1 (10^−9^ M) for glycoconjugate secretion (**d**). Data are mean ± SEM from three (**b**,**c**) and six (**d**) experiments). White triangles indicate individual data points. * shows significance above zero (basal). # shows significant difference between agonist and agonist + inhibitor.

Multiple SPMs that stimulate goblet cell secretion, also increase [Ca^2+^]_i_, in cultured rat conjunctival goblet cells [15,16,17,18,19]. Cultured goblet cells were incubated in Fura2/AM for one hour and stimulated with MaR2 (10^−10^ M–10^−8^ M), and as controls MaR1 at 10^−8^ M or histamine at 10^−5^ M. MaR2 increases [Ca^2+^]_i_ rat conjunctival goblet cells in a concentration-dependent manner (Figure 2a,b). MaR2-stimulated increase in [Ca^2+^]_i_, was 62.36 ± 12.22 nM (*p* = 0.007,) for 10^−10^ M, 93.48 ± 12.66 nM (*p* = 0.002), for 10^−9^ M and 189.20 ± 14.54 nM (*p* = 0.029) for 10^−8^ M, (Figure 2c; *n* = 3). The highest increase in peak [Ca^2+^]_i_ was triggered by MaR2 10^−8^ M; thus, this concentration was used in further experiments. MaR1 at 10^−8^ M and histamine at 10^−5^ M also increased [Ca^2+^]_i_.

Goblet cells were preincubated with BAPTA/AM (10^−4^ M) which chelates Ca^2+^_i_ as it is released by agonists. Cells were then stimulated with MaR2 (10^−8^ M) or MaR1 (10^−9^ M) and secretion measured (Figure 2d; *n* = 6). MaR2 stimulated secretion was completely blocked and MaR1 secretion partially, but significantly blocked.

The results from Figure 2 suggest that MaR2 increases [Ca^2+^]_i_ and this increase leads to secretion. 

### 2.3. Maresin 2 Activates the BLT1 Receptor, but Not the ALX/FPR 2 Receptor to Increase [Ca^2+^]_i_

The formyl peptide receptors (FPRs) are a family of receptors including three subtypes in mammals; FPR1, FPR2 and FPR3. The first described ligand of the receptor was a formylated peptide from Escherichia coli, which binds with high affinity [21]. The Lipoxin-receptor (ALX)/N-formyl-peptide receptor (FPR2) (ALX/FPR2)-receptor is a complex G-protein coupled receptor (GPCR) which is known to bind a variety of ligands, including proteins/peptides such as Annexin A1 and serum amyloid a (SAA), lipids like RvD1 and LxA_4_ and small molecules like compound 43 (C43) [19,22]. The ALX/FPR2 receptor is present in rat conjunctival goblet cells, confirmed both by western blot analysis and RT-PCR [19,23,24,25]. Annexin A1 (AnxA1), LxA4 and RvD1 have been found to increase [Ca^2+^]_i_ and stimulate secretion by binding to the receptor in rat conjunctival goblet cells [19,26,27]. Moreover, MaR1 is dependent on the ALX/FPR-2 receptor to increase [Ca^2+^]_i_ and to stimulate glycoconjugate secretion in conjunctival goblet cells [15]. Thus, we determined the role of the ALX/FPR2-receptor in MaR2-stimulated increase in [Ca^2+^]_i_. 

**Figure 3:**
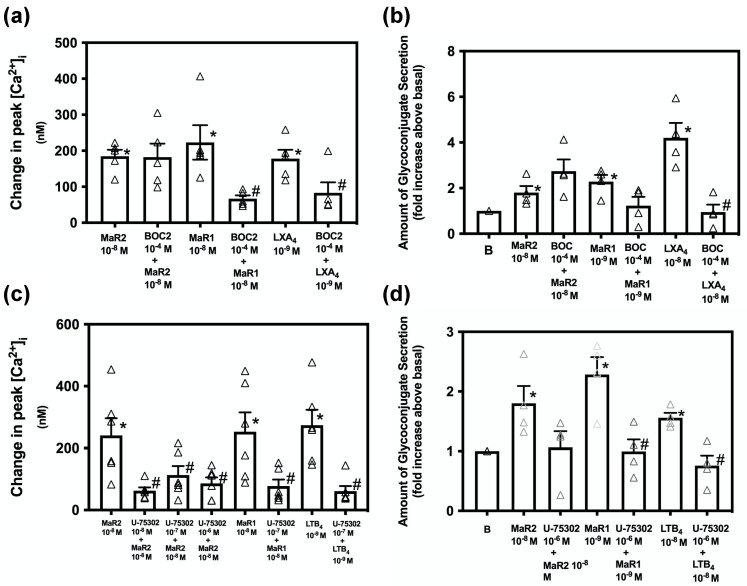
Inhibition of the BLT1, but not the ALX/FPR2 receptors acts on stimulation of [Ca^2+^]_i_ and secretion by MaR2. Goblet cells were treated with the ALX/FPR2 receptor inhibitor BOC2 (10^−4^ M) for 30 min and stimulated with MaR2 10^−8^ M (**a**,**b**), MaR1 10^−8^ M (**a**) MaR1 10^−9^ M (**b**), LXA_4_ 10^−9^ M (**a**) or LXA_4_ 10^−8^ M (**b**). Goblet cells were treated with the BLT1 receptor inhibitor U-75302 (10^−8^–10^−6^ M) for 30 min and stimulated with MaR2 10^−8^ M (**c**,**d**), MaR1 10^−8^ M (**c**), MaR1 10^−9^ M (**d**) or LTB_4_ 10^−9^ M (**c**) LTB_4_ 10^−8^ M (**d**). [Ca^2+^]_i_ was measured in (**a**,**c**); secretion in (**b**,**d**). Data are mean ± SEM of five (**a**), four (**b**), six (**c**) and four (**d**) experiments. White triangles indicate individual data points. * shows significance above basal. # shows significance between agonist and inhibitor + agonist.

Rat conjunctival goblet cells were incubated with the ALX/FPR2 inhibitor N-BOC-Phe-Leu-Phe-Leu-Phe (BOC2) (10^−4^ M) for 30 min prior to stimulation with MaR2 (10^−8^ M). MaR1 (10^−8^ M) and LXA_4_ (10^−9^ M) were used as positive controls [15,19]. Supplementary Figure S1 indicates changes in [Ca^2+^]_i_ over time. MaR2 significantly increased [Ca^2+^]_i_ to 184.66 ± 18.00 nM (*p* = 7× 10^−6^) while the MaR2 10^−8^ M treated with BOC2 increased [Ca^2+^]_i_ to 182.33 ± 37.50 nM (*p* = 0.96, Figure 3a; *n* = 5). MaR1 added alone significantly increased [Ca^2+^]_i_ to 223.30 ± 47.88 nM (Figure 3a; *n* = 5). BOC2 significantly decreased the MaR1 response to 66.98 ± 8.95 nM. LXA_4_-stimulated [Ca^2+^]_i_ increase was 178.10 ± 24.68 nM (Figure 3a; *n* = 5). In the presence of BOC2 the LXA_4_ response was significantly reduced to 83.13 ± 29.00. These results show that MaR2 does not activate the ALX/FPR2 receptor to increase [Ca^2+^]_i_, but the positive controls MaR1 and LXA_4_ do.

To explore the role of the ALX/FPR2-receptor in glycoconjugate secretion, goblet cells were incubated with BOC2 for 30 min prior to stimulation with MaR2 and the controls MaR1 and LXA_4_. MaR2 increased secretion 1.8 ± 0.3 -fold above basal (Figure 3b; *n* = 4). The MaR2 response was not significantly inhibited by BOC2, while secretion stimulated by the positive controls MaR1 was decreased and LXA_4_ was significantly inhibited by the ALX/FPR2 inhibitor. These data indicate that MaR2 does not utilize the ALX/FPR2-receptor to increase [Ca^2+^]_i_ or stimulate secretion.

LTB_4_ activates the GPCR receptor, BLT1, to cause chemotactic, pro-inflammatory actions [28]. SPMs including RvE1 and MaR1 also bind to this receptor, but are pro-resolving [15,29]. To examine if MaR2 is using the BLT1 receptor to increase [Ca^2+^]_i_, rat conjunctival goblet cells were treated with an inhibitor of the BLT1 receptor, U-75302 (10^−8^–10^−6^) for 30 min. MaR2 10^−8^ M significantly increased [Ca^2+^]_i_ to 240.96 ± 55.34 nM (*p* = 0.001, Figure 3c; *n* = 6). Preincubation with the BLT1 receptor inhibitor U-75302 caused a MaR2 stimulated [Ca^2+^]_i_ increase to only 83.06 ± 17.80 (*p* = 0.021), 113.05 ± 29.34 (*p* = 0.07) and 62.88 ± 10.55 (*p* = 0.01) (Figure 3c; *n* = 6) with 10^−8^ M, 10^−7^ M and 10^−6^ M inhibitor, respectively. The positive controls MaR1 at 10^−8^ M and LTB_4_ at 10^−9^ M increased [Ca^2+^]_i_ that was blocked by U-75302 (Figure 3c; *n* = 6). These results suggest that MaR2, similarly to the positive controls MaR1 and LXA_4_, is dependent on the BLT1 receptor to increase [Ca^2+^]_i_. 

To examine the dependency of MaR2- stimulate secretion on the BLT1 receptor, rat conjunctival goblet cells were treated with U-75302 (10^−6^ M) for 30 min before addition of MaR2. MaR2 increased secretion 1.8 ± 0.3 -fold above basal (Figure 3d; *n* = 4). MaR2 was not significantly inhibited by U-75302. Secretion stimulated by the positive controls MaR1 and LTB_4_ was significantly inhibited by U-75302. These data indicate that MaR2 uses the BLT1-receptor to increase [Ca^2+^]_i_, but not secretion.

### 2.4. Maresin 2 Does Not Inhibit Maresin 1 Stimulated Increase in [Ca^2+^]_i_, but Maresin 1 Inhibits Maresin 2-Stimulated [Ca^2+^]_i_

**Figure 4:**
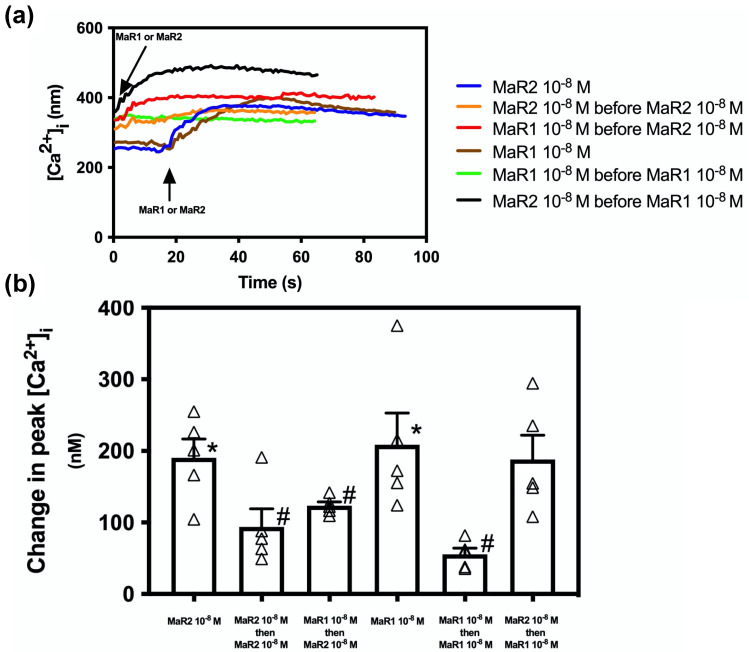
Maresin 2 does not inhibit Maresin 1-stimulated increase in [Ca^2+^]_i,_ but Maresin 1 does inhibit Maresin 2 stimulation. (**a**) shows changes in [Ca^2+^]_i_ with time, while (**b**) shows changes in peak of [Ca^2+^]_i_. Goblet cells were preincubated 2 min with either MaR2 (10^−8^ M) (blue line a, first bar b) or MaR1 (10^−8^ M) (brown line a, fourth bar b), then stimulated with MaR2 (10^−8^ M) (orange line a, second bar b) or MaR1 (10^−8^ M (green line a, fifth bar b), respectively or stimulated with MaR1 before MaR2 (red line a, third bar b) or MaR2 before MaR1 (black line a, (sixth bar b). Data are mean ± SEM of five (a and b) experiments. White triangles indicate individual data points. * shows significance above basal. # shows significance between MaR2 and MaR2 after MaR2 or MaR1, and between MaR1 and MaR1 after MaR2 or MaR1.

Both MaR1 and MaR2 are SPMs derived from the ω-3 fatty acid docosahexaenoic acid (DHA). To determine if the two MaRs desensitize each other and thus would activate the same receptor, we incubated rat conjunctival goblet cells two minutes with MaR2 (10^−8^ M) or MaR1 (10^−8^ M) alone. Then as a control to ensure that each of the MaRs can desensitize themselves, cells were stimulated with MaR2 10^−8^ M or MaR1 10^−8^ M followed by MaR2 or MaR1, respectively. Then MarR1 was added before MaR2 to determine if MaR1 desensitized MaR2 and Mar2 before MaR1 to determine if MaR2 desensitized MaR1. MaR2 caused an increase in peak [Ca^2+^]_i_ to 190.59 ± 21.78 (*p* = 3.38 × 10^−5^, Figure 4 a blue line and b first bar; *n* = 5). MaR2 addition before MaR2 caused an increase in peak [Ca^2+^]_i_ to 93.84 ± 25.20 that was significantly decreased compared to MaR2 alone (*p* = 0.031, Figure 4a orange line and Figure 4b second bar; *n* = 5). Addition of MaR1 before MaR2 caused an increase in peak [Ca^2+^]_i_ to 112.51 ± 7.02 that was significantly decreased compared to MaR2 alone ( *p* = 0.018, Figure 4 a red line and b third bar; *n* = 5). 

MaR1 (10^−8^ M) caused an increase in [Ca^2+^]_i_ to 208.58 ± 44.19 (*p* = 0.002, Figure 4a brown line and Figure 4b fourth bar; *n* = 5). Addition of MaR1 before MaR1 caused an increase to 55.41 ± 8.61 that was significantly decreased compared to MaR1 alone (*p* = 0.009, Figure 4a green line and Figure 4b fifth bar; *n* = 5). Addition of MaR2 before MaR1 caused an increase to 188.28 ± 26.07 that was not different from MaR1 alone (*p* = 0.58, Figure 4a black line and Figure 4b sixth bar; *n* = 5). 

These results show that when MaR2 activates its receptor first, MaR1 can also activate it, suggesting that MaR2 and MaR1 are activating different receptors, or overlapping areas on the same receptor. In contrast, when MaR1 activates its receptor first, MaR2 cannot activate it, suggesting that MaR1 and MaR2 activate the same receptor. 

### 2.5. Maresin 2 Increase in [Ca^2+^]_i_, but Not Secretion_,_ Is Independent of the PLC-Pathway in Rat Conjunctival Goblet Cells

**Figure 5:**
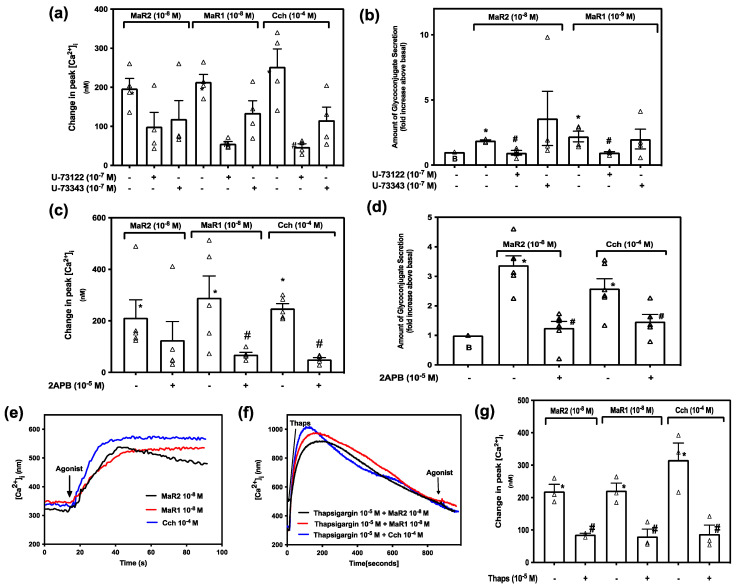
Maresin 2 increase in [Ca^2+^]_i,_ but not secretion_,_ is independent of the PLC-pathway in rat conjunctival goblet cells. Goblet cells were treated with vehicle, the active PLC inhibitor U-73122 or the inactive PLC inhibitor U-73343 both at 10^−7^ M for 30 min and stimulated with MaR2 (10^−8^ M), MaR1 (10^−8^ M) or Cch (10^−4^ M). (**a**) shows changes in peak [Ca^2+^]_i_ and (**b**) shows glycoconjugate secretion. Goblet cells were treated with vehicle or 2APB (10^−5^ M) and stimulated with MaR2 (10^−8^ M), MaR1 (10^−8^ M) or Cch (10^−4^ M). (**c**) shows changes in peak [Ca^2+^]_i_, and (**d**) shows glycoconjugate secretion. Goblet cells were stimulated with MaR2 (10^−8^ M), MaR1 (10^−8^ M) or Cch (10^−4^ M) alone or incubated with vehicle or thapsigargin (10^−5^ M) for 15 min and then stimulated with MaR2 (10^−8^ M), MaR1 (10^−8^ M) or Cch (10^−4^ M). (**e**,**f**) show changes in [Ca^2+^]_i_ with time, while (**g**) shows changes in peak of [Ca^2+^]_i_. Data are mean ± SEM of four (**a**), four (**b**), five (**c**), six (**d**), and three (**e**–**g**) experiments. White triangles indicate individual data points. * shows significance above basal. # shows significance between MaR2 and inhibitor then MaR2 or control and inhibitor then control.

The PLC pathway is activated in a variety of cellular processes, including exocytosis and fluid secretion [30]. This intracellular signaling pathway is essential for the function of MaR1 in rat conjunctival goblet cells [15]. To determine if MaR2 uses the same pathway components to increase [Ca^2+^]_i_ we treated rat conjunctival goblet cells for 30 min with the PLC inhibitor U-73122 (10^−7^ M) or its inactive control U-73343 (10^−7^ M) before stimulating with MaR2 (10^−8^ M) (Figure 5a; *n* = 4), or the positive controls MaR1 (10^−8^ M) (Figure 5a; *n* = 4) or Cch 10^−4^ M (Figure 5a; *n* = 4). Supplementary Figure S2 indicates changes in [Ca^2+^]_i_ over time. MaR2 10^−8^ M caused an increase in peak [Ca^2+^]_i_ to 196.87 ± 25.62 (*p* = 0.0003, Figure 5a; *n* = 4). Treatment with U-73122 or U-73343 followed by MaR2 caused an increase in peak [Ca^2+^]_i_ to 99.28 ± 36.77 (*p* = 0.072), and 118.95 ± 47.13 (*p* = 0.20), respectively, that were unchanged when compared to MaR2 stimulation. MaR1 and Cch stimulation, in contrast to that of MaR2, is dependent on PLC to increase [Ca^2+^]_i_ as their action on [Ca^2+^]_i_ was decreased by U-73122, but not by U-73343 (Figure 5a; *n* = 4). 

The action of PLC on MaR2-stimulated increase in glycoconjugate secretion was next investigated. MaR2 increased secretion 1.9 ± 0.1 -fold above basal (Figure 5b; *n* = 4). The response was significantly blocked by U73122 to 0.9 ± 0.2 (*p* = 0.003), but not by the inactive control U73343 (3.6 ± 2.1) (*p* = 0.44). MaR1- and Cch-stimulated increase in secretion were also significantly blocked by U73122, but not by U73343 (Figure 5b; *n* = 4). We conclude that MaR2 is dependent upon activation of the PLC pathway to stimulate glycoprotein secretion, but MaR2 does not use PLC to increase [Ca^2+^]_i_.

Activation of the PLC pathway produces IP_3_ which binds to its intracellular receptor on the ER causing release of Ca^2+^ from intracellular calcium stores increasing [Ca^2+^]_i_. To determine if MaR2 is dependent of the downstream molecules that activation of the PLC pathway produces, cells were treated with the IP_3_-receptor inhibitor 2APB (10^−5^ M) and then stimulated with MaR2 10^−8^ M. MaR1 (10^−8^ M) and Cch (10^−4^ M) were used as positive controls. MaR2 caused an increase in peak [Ca^2+^]_i_ to 212.28 ± 69.55 (*p*=0.016, Figure 5c; *n* = 5). Treatment with 2APB (10^−5^ M) did not alter the increase in peak [Ca^2+^]_i_ (125.37 ± 72.15; *p* = 0.41) compared with the action of MaR2 alone. In contrast treatment with 2-APB blocked the action of MaR1 and Cch on [Ca^2+^]_i_ (Figure 5c; *n* = 5). We conclude that the action of MaR2 is independent of the action of IP_3_ on its receptor to increase [Ca^2+^]_i_. 

The effect 2APB on MaR2-stimulated increase in glycoconjugate secretion was then explored. MaR2-increased secretion was 3.3 ± 0.5 -fold above basal (Figure 5d; *n* = 6). 2APB significantly decreased MaR2-stimulated response to 1.3 ± 0.2 -fold (*p* = 0.0002, Figure 5d; *n* = 6). The action of the positive control, Cch, on secretion was also significantly inhibited by 2APB (Figure 5d; *n* = 6). We conclude that the action of MaR2 on [Ca^2+^]_i,_ but not secretion, is independent of the action of IP_3_ on its receptor to increase [Ca^2+^]_i_.

To determine if MaR2 is using intracellular calcium stores to increase [Ca^2+^] by other mechanisms than PLC-IP_3_ pathway, we used the sarco/endoplasmic reticulum Ca^2+^-ATPase (SERCA) inhibitor thapsigargin. Thapsigargin blocks the uptake of Ca^2+^ into intracellular stores so that the cytoplasmic [Ca^2+^]_i_ increases by a passive leak from the ER. If an agonist uses the same intracellular Ca^2+^ store as thapsigargin, the increase in [Ca^2+^]_i_ by an agonist added after thapsigargin will be decreased. Conjunctival goblet cells were treated by thapsigargin (10^−5^ M) for 15 min that releases Ca^2+^ from the intracellular stores and then stimulated with MaR2 (10^−8^ M), or the positive controls MaR1 (10^−8^ M) or Cch (10^−4^ M). MaR2 increased [Ca^2+^]_i_ to a peak of 219.10 ± 21.63 nM (*p* = 0.00053, Figure 5e,g; *n* = 3). Treatment with thapsigargin caused a MaR2 increase in [Ca^2+^]_i_ to a peak of 85.68 ± 4.02 nM that was significantly decreased compared to MaR2 alone (*p* = 0.0037, Figure 5f,g; *n* = 3). A similar effect of thapsigargin was detected using MaR1 and Cch. Thus, MaR2 is dependent on a release of Ca^2+^ from intracellular calcium stores to increase [Ca^2+^]_i_.

MaR2 does not use PLC to increase [Ca^2+^]_i_ as neither the active PLC inhibitor nor the inhibitor of IP_3_ for its receptor on the ER blocked MaR2 increase in [Ca^2+^]_i._ MaR2, however, does appear to use PLC to stimulate secretion as the inhibitors tested are in agreement on their actions. The inhibitor of SERCA on the ER does block MaR2 increase in [Ca^2+^]_i,_ but this could belong to an as yet unidentified pathway. 

### 2.6. Maresin 2 Stimulated Increase in [Ca^2+^]_i_ Is Independent of Extracellular Ca^2+^ in Rat Conjunctival Goblet Cells

**Figure 6:**
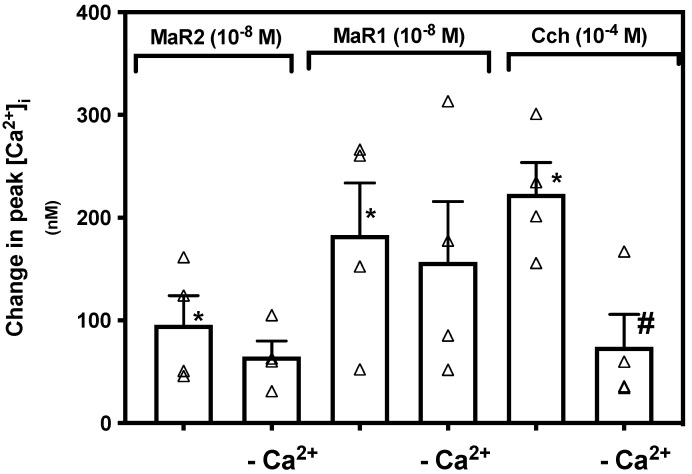
Maresin 2 stimulated increase in [Ca^2+^]_i_ is independent of extracellular Ca^2+^ in rat conjunctival goblet cells. Goblet cells were incubated with vehicle with or without CaCl_2_, then stimulated with MaR2 (10^−8^ M), or the controls MaR1 (10^−8^ M) and Cch (10^−4^ M). Figure shows changes in peak of [Ca^2+^]_i_. Data are mean ± SEM of four experiments. White triangles indicate individual data points. * shows significance above basal. # shows significance between agonist and extracellular Ca^2+^ removal then agonist.

In conjunctival goblet cells MaR1 is not dependent on extracellular Ca^2+^ to increase [Ca^2+^]_i_ [15]. To determine if MaR2 is dependent on influx of extracellular Ca^2+^, we incubated rat conjunctival goblet cells in vehicle with or without CaCl_2_ (1.0 mM). Supplementary Figure S3 indicates changes in [Ca^2+^]_i_ over time. MaR2 with CaCl_2_ increased [Ca^2+^]_i_ to a peak of 95.66 ± 28.34 nM (*p* = 0.015, Figure 6; *n* = 4), while MaR2 without CaCl_2_ increased [Ca^2+^]_i_ to a peak of 64.74 ± 15.25 nM, a non-significant decrease (*p* = 0.37). A similar finding was detected for MaR1 (10^−8^ M) (Figure 6; *n* = 4). In contrast [Ca^2+^]_i_ stimulated by Cch (10^−4^ M) was significantly decreased in the absence of extracellular Ca^2+^ (Figure 6; *n* = 4). We conclude that MaR2, similarly to MaR1, is independent of influx of extracellular Ca^2+^ to increase [Ca^2+^]_i_, but Cch is not.

### 2.7. Maresin 2 Increases [Ca^2+^]_i_ and Stimulates Secretion by Activation of Protein Kinase C 

**Figure 7:**
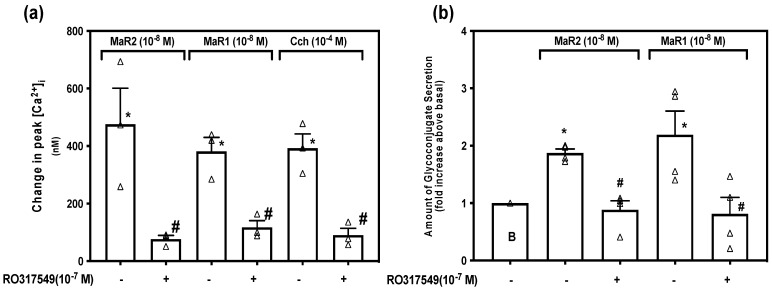
Maresin 2 increases [Ca^2+^]_i_ and stimulates secretion by activation of protein kinase C (PKC). Goblet cells were incubated with RO317549 (10^−7^ M) for 30 min, then stimulated with MaR2 (10^−8^ M) or the positive controls MaR1 (10^−8^ M) or Cch (10^−4^ M) and then [Ca^2+^]_i_ and secretion were measured. (**a**) shows change in peak [Ca^2+^]_i_, while (**b**) shows -fold increase in glycoconjugate secretion. Data are mean ± SEM of three (**a**) and four (**b**) experiments. White triangles indicate individual data points. * shows significance above basal. # shows significance between agonist and inhibitor then agonist.

Protein kinase C (PKC) is activated by diacylglycerol (DAG) produced by activation of PLC when PLC also produces IP_3_. PKC can also be activated by other signaling pathways. To determine if MaR2 is dependent on PKC to increase [Ca^2+^]_i_ we incubated conjunctival goblet cells with the PKC inhibitor RO317549 (10^−7^ M) for 30 min, then stimulated with MaR2 (10^−8^ M), or the positive controls MaR1 (10^−8^ M) and Cch (10^−4^ M). Supplementary Figure S4 indicates changes in [Ca^2+^]_i_ over time. MaR2 increased [Ca^2+^]_i_ to a peak of 475.49 ± 125.41 nM (*p* = 0.019, Figure 7a; *n* = 3), while treatment with RO317549 significantly decreased [Ca^2+^]_i_ to a peak of 76.67 ± 12.84 nM (*p* = 0.034, Figure 7a; *n* = 3). Similar results were obtained with MaR1 and Cch (Figure 7a; *n* = 3). Thus, MaR2 is dependent on PKC to increase [Ca^2+^]_i_, as are MaR1 and Cch.

The effect of RO317549 on MaR2-stimulated increase in glycoconjugate secretion was determined after a 30 min incubation with RO317549 (10^−7^ M). MaR2 increased secretion 1.9 ± 0.1 -fold above basal (*p* = 1.44 × 10^−5^, Figure 7b; *n* = 4) and MaR2 stimulation was significantly blocked by RO317549 to 0.9 ± 0.2 -fold above basal (*p* = 0.001). The positive control, MaR1, increased secretion to 2.2 ± 0.8 -fold above basal (*p* = 0.03) and preincubation with RO317549 significantly decreased the response to 0.8 ± 0.2 -fold above basal (*p* = 0.03). Both MaR2 and MaR1 are dependent upon PKC to increase [Ca^2+^]_i_ and stimulate secretion.

### 2.8. Maresin 2 Has Different Dependency on Phospholipase D (PLD) Compared to Phospholipase A_2_ (PLA_2_) to Increase [Ca^2+^]_i_ and Stimulate Secretion in Rat Conjunctival Goblet Cells

**Figure 8:**
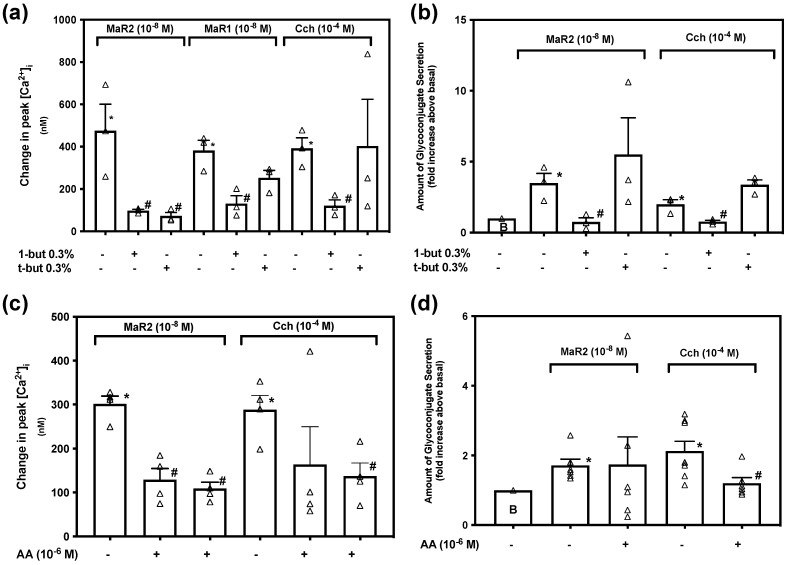
Maresin 2 has different dependency on Phospholipase D (PLD) compared to Phospholipase A_2_ (PLA_2_) to increase [Ca^2+^]_i_ and stimulate secretion in rat conjunctival goblet cells. Goblet cells were preincubated with the PLD inhibitor 0.3% 1-butanol or the inactive analog 0.3% t-butanol for 15 min and then stimulated with MaR2 (10^−8^ M), MaR1 (10^−8^ M) or Cch (10^−4^ M) to measure the change in peak [Ca^2+^]_i_ (**a**), or secretion (**b**). Goblet cells were preincubated with the PLA_2_ inhibitor aristolochic acid 10^−5^ M or 10^−6^ M for 30 min and stimulated with MaR2 (10^−8^ M) or Cch (10^−4^ M) to measure the change in peak [Ca^2+^]_i_ (**c**), or secretion (**d**). Data are mean ± SEM of three (**a**), three (**b**), four (**c**) and six (**d**) experiments. White triangles indicate individual data points. * shows significance above basal. # shows significance between agonist and inhibitor followed by agonist.

Activation of Phospholipase D (PLD) is controlled by multiple mechanisms, activates distinct pathways and is important in cellular functioning [31]. To explore if MaR2 uses PLD to increase [Ca^2+^]_i_ we used the PLD-inhibitor 1-butanol (1-but) at 0.3% and the inactive control t-butanol (t-but) at 0.3%. Supplementary Figure S5 indicates changes in [Ca^2+^]_i_ over time. MaR2 (10^−8^ M) caused an increase in [Ca^2+^]_i_ to a peak of 475.49 ± 125.41 nM (*p* = 0.019, Figure 8a; *n* = 3). MaR2 (10^−8^ M) after 1-butanol (active analog) increased [Ca^2+^]_i_ to a peak of 97.89 ± 6.41 nM that was significantly decreased compare to MaR2 alone (*p* = 0.040). MaR2 (10^−8^ M) added after t-butanol (inactive analog) increased [Ca^2+^]_i_ to a peak of 72.40 ± 17.05 nM that was significantly decreased from MaR2 alone (*p* = 0.033). For the positive controls, MaR1 and carbachol stimulation of peak in [Ca^2+^]_i_ was decreased by 1-butanol, but not by t-butanol (Figure 8a; *n* = 3). Although the peak in [Ca^2+^]_i_ was reduced by 1-butanol, we cannot definitively conclude that the action of MaR2 is dependent on PLD, due to inhibition by the inactive control t-butanol. In contrast, MaR1 and Cch stimulation were dependent on the activation of PLD.

To explore the role of PLD in MaR2-stimulated glycoconjugate secretion, rat conjunctival goblet cells were incubated with 1-butanol or t-butanol then stimulated with MaR2. MaR2 increased secretion 3.5 ± 0.7 -fold above basal (*p* = 0.007, Figure 8b; *n* = 3). 1-butanol (*p* = 0.02), but not t-butanol (*p* = 0.50), significantly inhibited MaR2-stimulated secretion. The positive controls, MaR1 and Cch stimulated glycoconjugate secretion above basal and 1-butanol, but not t-butanol, significantly decreased the response for each agonist. This indicates that MaR2 utilizes PLD to stimulate glycoconjugate secretion, as do MaR1 and carbachol.

To examine if MaR2 is dependent on PLA_2_ to increase [Ca^2+^]_i_ we used the PLA_2_-inhibitor Aristolochic Acid (AA). Supplementary Figure S5 indicates changes in [Ca^2+^]_i_ over time. MaR2 (10^−8^ M) caused an increase in [Ca^2+^]_i_ to a peak of 301.53 ± 17.7 nM (*p* = 0.05, Figure 8c; *n* = 4). When incubated with AA 10^−5^ M or AA 10^−6^ M, MaR2 caused an increase in [Ca^2+^]_i_ to a peak of 108.77 ± 14.83 nM or 129.09 ± 25.71 nM, respectively values that were significantly decreased from MaR2 alone (*p* < 0.001 for AA 10^−5^ M and *p* = 0.001 for AA 10^−6^ M). The action of Cch on peak increase in [Ca^2+^]_i_ was blocked by AA at 10^−5^ M (Figure 8c; *n*= 4). Thus MaR2 activates PLA2 to increase [Ca^2+^]_i_.

To determine if the action of MaR2 is dependent on PLA_2_ to stimulate glycoconjugate secretion, conjunctival goblet cells were preincubated with AA. MaR2 increased secretion 1.7 ± 0.2 -fold above basal (*p* = 0.003, Figure 8d; *n*=6). When incubated with AA at 10^−5^ M MaR2 increased secretion to 1.7 ± 0.8 -fold above basal (*p* = 0.97), not a significantly different value from stimulation with MaR2 alone. The increase in glycoconjugate secretion stimulated by the positive control, Cch was significantly decreased by AA (Figure 8d; *n* = 6). This indicates that MaR2 uses PLA_2_ to increase [Ca^2+^]_i_, but not to stimulate glycoconjugate secretion.

### 2.9. Maresin 2 Uses Protein Kinase A to Increase [Ca^2+^]_i_ and Stimulate Secretion in Rat Conjunctival Goblet Cells

**Figure 9:**
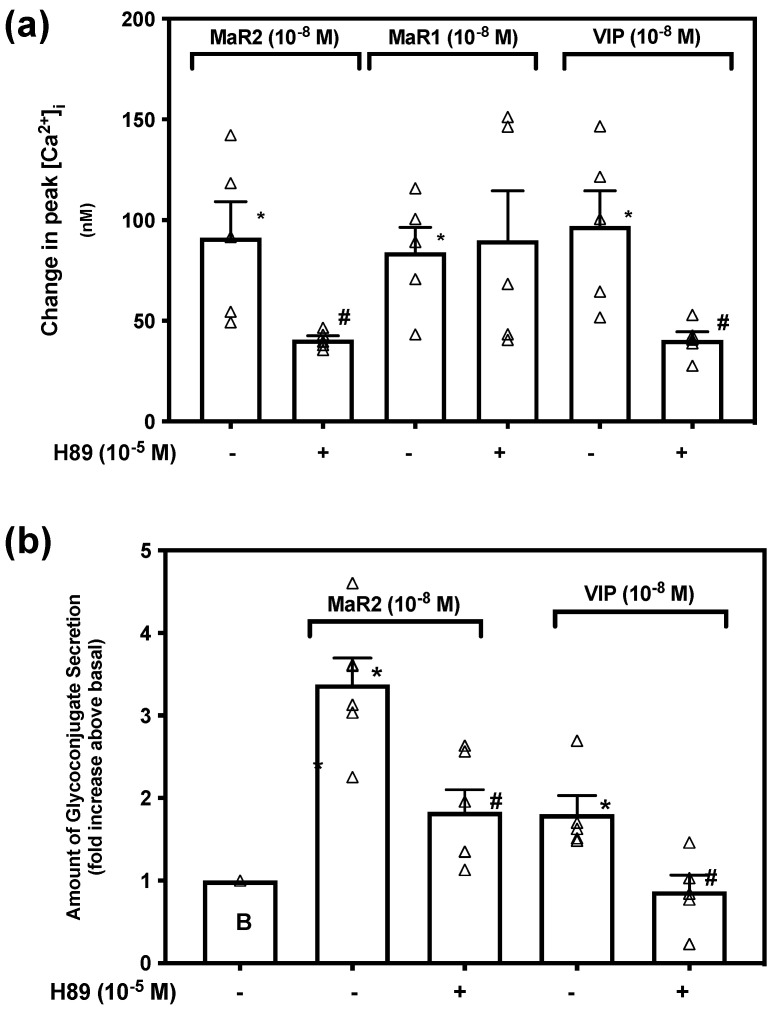
Maresin 2 uses protein kinase A to increase [Ca^2+^]_i_ and stimulate secretion in rat conjunctival goblet cells. Goblet cells were incubated with the protein kinase A (PKA) inhibitor H89 (10^−5^ M) for 30 min and then stimulated with MaR2 (10^−8^ M) or the positive controls MaR1 (10^−8^ M) and VIP (10^−8^ M) to measure the change in peak [Ca^2+^]_i_ (**a**)**,** or secretion (**b**). Note that MaR1 was not used as an agonist in (**b**). Data are mean ± SEM of five (**a**) and six (**b**) experiments. White triangles indicate individual data points. * shows significance above basal. # shows significance between agonist and inhibitor followed by agonist.

When a ligand activates Gαs, adenylyl cyclase (AC) catalyzes ATP to cAMP that in turn stimulates the activity of cAMP dependent protein kinase A (PKA). This is one among a variety of functions of cAMP [32]. To explore if MaR2 uses PKA, we incubated rat conjunctival goblet cells with the PKA-inhibitor H89 (10^−5^ M) for 30 min prior to stimulation with MaR2 (10^−8^ M), or the positive controls VIP (10^−8^ M) and MaR1 (10^−8^ M). Supplementary Figure S6 indicates changes in [Ca^2+^]_i_ over time. MaR2 increased [Ca^2+^]_i_ to a peak of 91.27 ± 17.97 nM (*p* = 0.00010, Figure 9a; *n* = 5). Incubation with H89 increased [Ca^2+^]_i_ to a peak of 40.66 ± 1.94 nM that was different from MaR2 alone (*p* = 0.023). The action of MaR1 was not inhibited by H89, but of VIP was blocked (Figure 9a; *n* = 5). This indicates that MaR2 activates PKA to increase [Ca^2+^]_i_.

To determine the dependency of MaR2 on PKA to stimulate glycoconjugate secretion, conjunctival goblet cells were incubated with H89 (10^−5^ M) 30 min prior to addition of MaR2. MaR2 stimulated secretion to 3.4 ± 0.5 -fold above basal (*p* = 2.2 × 10^−5^, Figure 9b; *n* = 6). Incubation with H89 significantly decreased MaR2-stimulated secretion to 1.8 ± 0.3 -fold above basal (*p*=0.004). Secretion stimulated by the positive control VIP was also significantly inhibited by H89. These data indicate that MaR2, but not MaR1, is dependent on activation of PKA to increase [Ca^2+^]_i_ and glycoconjugate secretion.

### 2.10. Maresin 2 Inhibits Histamine-, but Not LTB_4_-Stimulated Increase in [Ca^2+^]_i_ and Histamine-Stimulated Glycoconjugate Secretion in Rat Conjunctival Goblet Cells

**Figure 10:**
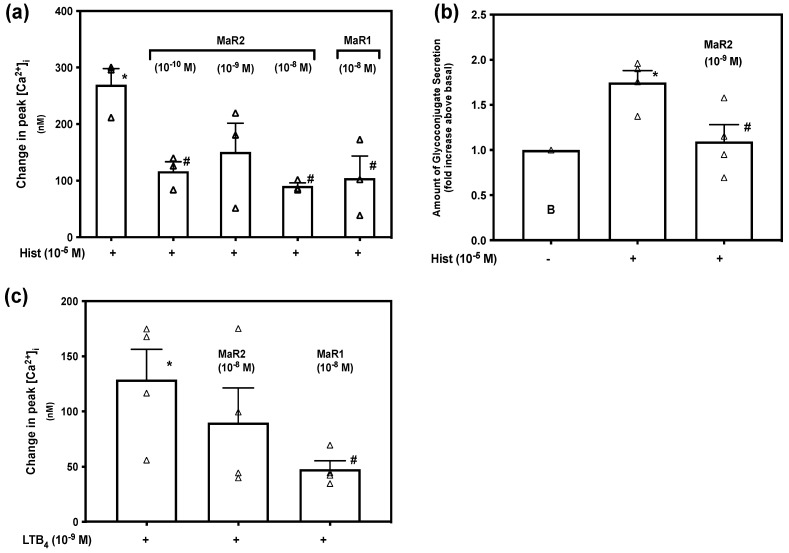
Maresin 2 inhibits histamine-, but not LTB_4_-stimulated increase in [Ca^2+^]_i_ and histamine-stimulated glycoconjugate secretion in rat conjunctival goblet cells. Goblet cells were incubated with MaR2 (10^−8^ M) and then stimulated with histamine (10^−5^ M). Goblet cells were incubated with MaR2 (10^−10^ to 10^−8^ M) or MaR1 (10^−8^ M) for 30 min, then stimulated with histamine (10^−5^ M). Changes in in peak [Ca^2+^]_i_ are shown in (**a**) and changes in glycoconjugate secretion are shown in (**b**). Goblet cells were incubated with MaR2 (10^−8^ M) or MaR1 (10^−8^ M) for 30 min, then stimulated with LTB_4_ 10^−9^ M. Changes in peak [Ca^2+^]_i_ are shown in (**c**). Data are mean ± SEM of three (**a**) and four (**b**) and four (**c**) experiments. White triangles indicate individual data points. * shows significance above basal. # shows significance between MaR2 or MaR1 treatment and histamine (**a**,**b**) or LTB_4_ (**c**) and histamine (**a**,**b**) or LTB_4_ (**c**) alone.

Histamine has a key role in inflammatory allergic diseases, and is mainly secreted by mast cells and basophils. Histamine uses four receptors (H_1_–H_4_), causing vasodilatation, and vascular permeability contributing to inflammation [33]. In rat conjunctival goblet cells, histamine increases [Ca^2+^]_i_ and stimulates glycoconjugate secretion [34]. Other SPMs, including MaR1, inhibit histamine-stimulated increase [Ca^2+^]_i_ and glycoconjugate secretion [15,16]. Supplementary Figure S7 indicates changes in [Ca^2+^]_i_ over time. Histamine caused an increase in [Ca^2+^]_i_ to a peak of 269.47 ± 28.92 nM (*p* = 0.00074, Figure 10a; *n* = 3). When incubated with MaR2 for 30 min before addition of histamine, the increase in peak [Ca^2+^]_i_ was attenuated to 116.79 ± 16.80 nM by MaR2 at 10^−10^ M (*p* = 0.010), to 150.66 ± 50.78 nM by MaR2 at 10^−9^ M (*p* = 0.11) and to 90.73 ± 5.55 nM by MaR2 10^−8^ M (*p*=0.0040). The positive control MaR1 at 10^−8^ M also blocked the histamine stimulated increase in [Ca^2+^]_i_. We conclude that MaR2 similarly to MaR1 inhibits the histamine stimulated increase in [Ca^2+^]_i_.

Dartt et al. demonstrated that histamine stimulates an increase in [Ca^2+^]_i_ and glycoconjugate secretion in rat conjunctival goblet cells [34]. To investigate if MaR2 inhibits histamine stimulated glycoconjugate secretion, rat conjunctival goblet cells were preincubated with MaR2 (10^−8^ M) for 30 min prior to stimulation with histamine (10^−5^ M). Histamine stimulated secretion to 1.75 ± 0.13 above basal (*p* = 0.001, Figure 10b; *n* = 4). Preincubation with MaR2 decreased the response to 1.09 ± 0.19 above basal (*p* = 0.029). This indicates that MaR2 inhibits histamine stimulated glycoconjugate secretion.

The leukotriene LTB_4_ is a chemoattractant involved in inflammation and immune response and activates inflammatory cells [28]. LTB_4_ binds to the BLT1 receptor and to the ALX/FPR2-receptor [35]. To determine if MaR2 and MaR1 act on LTB_4_-stimulated increase in [Ca^2+^]_i_, we pre-incubated rat conjunctival goblet cells with MaR2 (10^−8^ M) or MaR1 (10^−8^ M) for 30 min, then stimulated with LTB_4_ (10^−9^ M). Supplementary Figure S7 indicates changes in [Ca^2+^]_i_ over time. LTB_4_ caused an increase in [Ca^2+^]_i_ to a peak of 128.71 ± 27.51 nM (*p*=0.0034, Figure 10c; *n*=4). Incubation with MaR2 caused a LTB_4_-stimulated increase in [Ca^2+^]_i_ to a peak of 89.62 ± 31.53 nM (*p*=0.39) that was not different from stimulation by LTB_4_ alone. Incubation with MaR1 significantly decreased the LTB_4_-stimulated increase in [Ca^2+^]_i_ to a peak of 47.65 ± 7.65 nM (*p*=0.030). We conclude that MaR2 does not inhibit LTB_4_-stimulated increase in [Ca^2+^]_i_, while MaR1 does. 

## 3. Discussion

**Figure 11:**
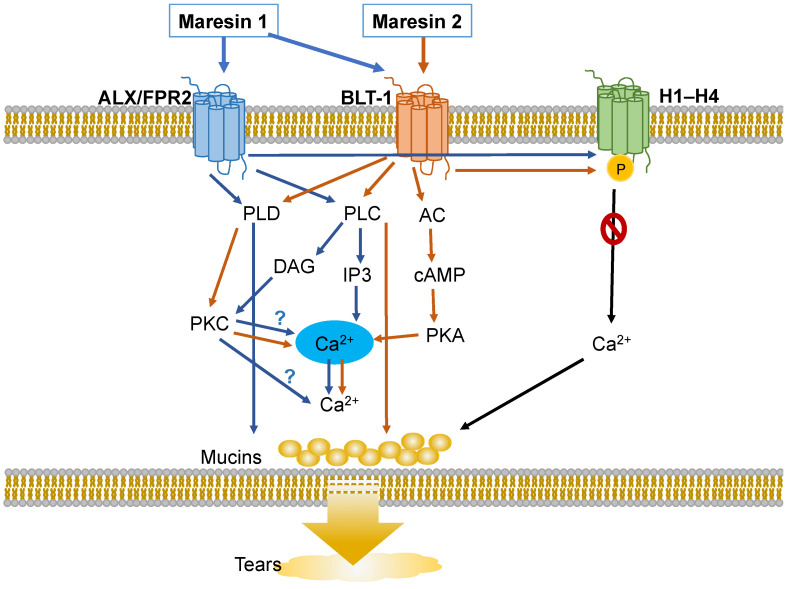
Schematic diagram of signaling pathways activated by Maresin 2 (MaR2) (orange arrows) compared to the pathways activated by Maresin 1 (MaR1) (blue arrows). MaR2 activates the BLT1 receptor activating PLD, AC, PLA_2,_ and PLC. PLD and AC activate downstream molecules that increase [Ca^2+^]_i_ causing glycoprotein secretion. PLA_2_ and the PLC pathway stimulates glycoprotein secretion by another unknown mechanism than increasing [Ca^2+^]_i_.

In the present study we showed that MaR2 activates rat conjunctival goblet cells through an increase in [Ca^2+^]_i_ that stimulates secretion and blocks overproduction of mucin stimulated by histamine, an allergic mediator. Both of these actions are used by MaR2 to maintain homeostasis in both health and disease (Figure 11). MaR2 uses the BLT1 receptor to increase [Ca^2+^]_i_ by activation of the cAMP-dependent PKA, PLD, PLC-PKC, and PLA_2_, but not the PLC-IP3, signaling pathways. None of the inhibitors of the signaling components, however, blocked MaR2-stimulated increase in [Ca^2+^]_i_ completely, indicating that multiple pathways/receptors could be involved in cellular activation. Pre-incubation with thapsigargin completely decreased MaR2-stimulated [Ca^2+^]_i_ increase, suggesting that activated signaling pathways caused a release of Ca^2+^ from intracellular calcium stores. Similarly to other SPMs, such as MaR1, LXA_4_, RvD1, RvD2, and RvE1, MaR2 regulates [Ca^2+^]_i_ and secretion including MUC5AC in rat conjunctival goblet cells [15,17,18,19,36]. These actions likely contribute to optimal tear film function under normal, physiological conditions. MaR2 also prevents the overproduction of mucin stimulated by histamine in ocular allergy. Thus MaR2 maintains homeostasis of tear film mucin in both health and disease. 

Although being of similar chemical structure, MaR2 and MaR1 activate different receptors. MaR2 to date only uses the BLT1 receptor and uses it only to increase [Ca^2+^]_i_. We found that MaR1 uses the BLT1- and the ALX/FPR2 receptor to increase [Ca^2+^]_i_, but only the BLT1 receptor to stimulate secretion. Treatment with MaR1 desensitizes MaR2, while preincubation with MaR2 does not affect the MaR1 response. There are several possible mechanisms that might explain how MaR1 attenuates MaR2 response. A possible mechanism of inhibition of MaR2 actions by MaR1 is through activation of the ALX/FPR2 receptor or through other receptors, including the newly identified LGR6 receptor for MaR1 that MaR2 does not stimulate [37]. It should be noted, however, that LGR6 was found in human, but not yet in rat, tissue. Furthermore, MaR1 may attenuate MaR2 by interacting with an overlapping or different region of the BLT1 receptor than MaR2 binds to. To support this hypothesis, we found that MaR1 decreases LTB_4_ induced increase in [Ca^2+^]_i_, while MaR2 does not. We suggest that MaR1, but not MaR2, may attenuate LTB_4_- and MaR2-dependent BLT1 responses by activating a protein kinase that phosphorylates the BLT1 receptor and counter-regulates it. MaR1, but not MaR2, could contribute to resolution of leukotriene-stimulated inflammation in ocular surface disease.

The BLT1 receptor is activated by the pro-inflammatory chemoattractant LTB_4_ [38]. We found that MaR1 was dependent on the BLT-1 receptor to increase [Ca^2+^]_i_, and to stimulate glycoconjugate secretion, while MaR2 was only dependent on the BLT1 receptor to increase [Ca^2+^]_i_. The fact that the pro-inflammatory mediator LTB_4_ and the pro-resolving mediators MaR1 and MaR2 are using the same receptor is an example of biased agonism. Biased agonism is when different ligands bind to a receptor to activate different signal transduction pathways, a phenomenon also found in receptors such as ALX/FPR2 [39]. The BLT1 receptor is a GPCR primarily known to couple to the inhibitory protein of the adenylyl cyclase, G_i_, and the stimulatory protein G_q_, the latter of which activates PLC, ultimately inducing chemotaxis [40]. We found that MaR2 can activate the BLT1 receptor, while also increase cAMP levels and stimulate PKA, which are activated by the protein G_s_. BLT1 does not couple to G_s_ and does not activate adenylyl cyclase suggesting that MaR2 could activate another receptor in rat conjunctival goblet cells to perform its actions. In support of this suggestion another SPM derived from DHA, RvE1, is known to bind to both the ChemR23 and BLT1 receptors. A central role for BLT1 in SPM functioning in rat conjunctival goblet cells is emerging [29].

Consistent with MaR2 and MaR1, interacting with different receptors or different sites on the same receptor, these SPMs differ in the use of the cAMP/PKA signaling pathway. MaR2, but not MaR1, increases cAMP levels and activates PKA to increase [Ca^2+^]_i_ and stimulate secretion. The only other SPM published to date in rats to use cAMP and PKA to increase in [Ca^2+^]_i_ and stimulate secretion is RvD2 [36]. Interestingly, in human immune cells MaR1 uses LGR6 to increase cAMP levels and activate PKA. RvD2 in rat and human conjunctival goblet cells uses the GPR18 receptor that activates adenylyl cyclase, to increase cAMP levels and activate PKA. Activation of PKA by itself stimulates secretion, but also increases [Ca^2+^]_i_ by interacting with the IP_3_ receptors on intracellular Ca^2+^ stores, likely on endoplasmic reticulum. Vasoactive intestinal peptide (VIP) is a parasympathetic neurotransmitter that like MaR2 and RvD2 stimulates PKA [41]. VIP activates the VPAC1 and the VPAC2 receptors causing activation of adenylyl cyclase that increases levels of cAMP, ultimately activating PKA. The activated PKA increases [Ca^2+^]_i_ through a mechanism that is similar to that used by RvD2 and slightly different from that used by MaR2. The difference is that VIP and RvD2 stimulate PLC activity to produce IP_3_. IP_3_ then binds with its receptors on the ER to release Ca^2+^ and cAMP that interacts with the IP_3_ receptors to increase Ca^2+^. In contrast, MaR2 does not activate PLC to produce IP_3._ Thus IP3 receptors are not involved in the action of MaR2. MaR2 activation of PKA would then increase [Ca^2+^]_i_ by a different mechanism than RvD2. Further studies are warranted to determine the specifics of the MaR2 cAMP-dependent actions and to compare them with those of RvD2 and VIP. 

In spite of MaR2 and MaR1 interacting with different receptors and activating different signaling pathways, MaR2 and MaR1 both use several similar Ca^2+^-dependent signaling pathways. First, both agonists increase [Ca^2+^]_i_ by release of intracellular Ca^2+^ stores, confirmed by inhibition of secretion when the [Ca^2+^]_i_ was decreased by the Ca^2+^ chelator BATPA/AM and when MaR2 and MaR1 stimulated increase in [Ca^2+^]_i_ was blocked by the SERCA inhibitor thapsigargin that depletes intracellular Ca^2+^ stores [15]. There are three main signaling pathways that SPMs use to increase [Ca^2+^]_i_ and stimulate secretion in conjunctival goblet cells PLA_2_, PLD and PLC. Neither MaR2 nor MaR1 activate PLA_2_ to increase [Ca^2+^]_i_ but MaR2 uses it to stimulate secretion. Both MaR2 and MaR1 activate PLD to increase [Ca^2+^]_i_ and stimulate secretion, although the negative control for MaR2 and PLD increase in [Ca^2+^]_i_ was also inhibitory. MaR2 and MaR1 both activate components of the PLC pathway. MaR2 and MaR1 activate PLC, but only MaR1 uses the downstream molecule IP_3_R and only MaR2-stimulated secretion is dependent on these components. Both MaR2 and MaR1 activated PKC to increase [Ca^2+^]_i_ and stimulate secretion. Surprisingly, both MaR2- and MaR1- stimulated increase in [Ca^2+^]_i_ are independent of extracellular Ca^2+^. Thus, the PLC pathway has some differences between MaR2 and MaR1 activation, especially in the targets of PLC activation. Whereas MaR1 stimulates the increase in [Ca^2+^]_i_ and secretion by the well-known PLC pathway that produces IP_3_ that releases Ca^2+^ from intracellular stores and produces DAG to activate PKC to stimulate secretion, MaR2 only uses these processes to stimulate secretion. As MaR2 and MaR1 activate the PLD pathway, they could use PLD to activate PKC via an increase in Ca^2+^. In contrast, MaR2 does not use PLC to increase Ca^2+^ and activate PKC, while MaR1 does. As there are multiple PKC isoforms in conjunctival goblet cells some of which are Ca^2+^ -dependent and Ca^2+^-independent PKC isoforms, MaR2 and MaR1 may be activating different PKC isoforms to stimulate secretion [42]. Identification of a MaR2-specific receptor and a more detailed investigation of the components of the signaling pathways could clarify some of the differences between MaR2 and MaR1 and their use of signaling pathways in conjunctival goblet cells.

A common type of chronic inflammation on the ocular surface is ocular allergy, a disease initiated by an allergic stimulus. Inflammatory ocular diseases usually cause hypersecretion of mucins. One of the central stimulatory mediators causing hypersecretion in allergic diseases is histamine [7]. When rat conjunctival goblet cells are preincubated with MaR2 before stimulation with histamine, the increase in both [Ca^2+^]_i_ and secretion decrease. This suggests that MaR2 can block the inflammatory effect of histamine on goblet cells decreasing mucin secretion. Many other SPMs, including LXA_4_, RvD1, RvE1, and MaR1, similarly counter-regulate the effect of histamine in cultured rat conjunctival goblet cells [15,16,43,44]. Ours findings herein support a role of MaR2, in homeostasis by stimulating goblet cell secretion in health and decreasing overproduction in diseases such as ocular allergy. 

Information about the function of MaR2 in disease in other organs is limited. MaR2 limits polymorphonuclear neutrophil (PMN) entry during inflammation and stimulates phagocytosis, similar to the actions of MaR1 [11,12]. Furthermore, the anti-inflammatory, pro-resolving, and anti-atherosclerotic effects of MaR2 might be beneficial in diseases such as myocardial infarction and acute and chronic heart failure [11,45]. MaR2 is likely to be active in many additional diseases and tissues.

We conclude that MaR2 and MaR1 stimulate conjunctival goblet cell function especially secretion, by activating different, but overlapping GPCR and signaling pathways, and furthermore counter-regulate histamine stimulated increase in [Ca^2+^] _i_. Thus, MaR2 and MaR1 play a role in maintaining the ocular surface and tear film homeostasis in health and disease. As MaR2 and MaR1 each modulate conjunctival goblet cell function, they each may have potential as novel, but differing, options for treatment of ocular surface inflammatory diseases including allergic conjunctivitis and dry eye disease.

## 4. Materials and Methods

### 4.1. Materials

RPMI-1640 cell culture medium, penicillin/streptomycin and L-glutamine were purchased from Lonza (Walkerville, IL, USA). Fetal bovine serum (FBS) was ordered from Atlanta Biologicals (Norcross, GA, USA). MaR2 and MaR1 were purchased from Cayman Chemical (Ann Arbor, MI, USA), stored in an ethanol solution at −80 °C. The solution was diluted immediately before use in Krebs-Ringer bicarbonate buffer with HEPES (KRB-HEPES, 119 mM NaCl, 4.8 mM KCl, 1.0 mM CaCl_2_, 1.2 mM MgSO_4_, 1.2 mM KH_2_PO_4_, 25 mM NaHCO_3_, 10 mM HEPES, and 5.5 mM glucose (pH 7.40–7.45)) to the desired concentrations and added to the cells. UEA-1 was obtained from Sigma-Aldrich (St. Louis, MO, USA). Vasoactive intestinal peptide (VIP), U73122 and U73343 were purchased from Tocris Bioscience (Ellisville, MO, USA). Histamine, carbachol (CCh), 2APB, 1-butanol (1-but) and tert-butanol (t-but) were obtained from Sigma-Aldrich (St. Louis, MO, USA). Fura-2/AM was purchased from Life Technologies (Grand Island, NY, USA). *n*-BOC-Phe-Leu-Phe-Leu-Phe (BOC2) was ordered from GenScript (Piscatawy, NJ, USA). BLT1 inhibitor U-75302 and LTB_4_ were ordered from Cayman Chemical (Ann Arbor, MI, USA). Lipoxin A_4_, H89, thapsigargin and RO-317549 were ordered from EMD Millipore (Billerica, MA, USA).

### 4.2. Animals

Male albino Sprague-Dawley rats from 4–6 weeks old (Taconic Farms, Germantown, NY, USA) were anesthetized with CO_2_ for 5 min, decapitated, and the bulbar and forniceal conjunctival epithelia removed from both eyes. All experiments were in accordance with the US Department of Health and Human Services Guide for the Care and Use of Laboratory Animals and were approved by the Schepens Eye Research Institute Animal Care and Use of Committee.

### 4.3. Cell Culture

Goblet cells were cultured from male albino Sprague-Dawley rat conjunctiva. The conjunctival tissue was cut into pieces that were placed in 6 well plates with 0.5 mL RPMI 1640 medium supplemented with 10% FBS, 2mM L-glutamine and 100 mcg/mL penicillin-streptomycin. The cells prepared for secretion experiments were plated in 24 well plates. RPMI media was changed every second day, and 2 mL media were used in each well. Cultured goblet cells were identified periodically by staining with anti-cytokeratin 7, anti-MUC5AC and the lectin UEA-1 directly conjugated to fluorophore. The cells were trypsinized and transferred to Ca^2+^ or secretion dishes 24 h before the experiments were performed.

### 4.4. Measurement of [Ca^2+^]_i_

Cultured rat conjunctival goblet cells were transferred after trypsinization to 35-mm glass bottom dishes and incubated in 37.0 °C overnight. The cells were then incubated in 37.0 °C for 1 h with KRB-HEPES containing 0.5% BSA, 0.5 mM fura2/AM, 250 mM Sulfinpyrazone and 8 mM Pluronic acid F127. [Ca^2+^] was measured with a ratio imaging system (InCytIm2; intracellular imaging, Cincinnati, OH, USA) using wavelengths of 340 and 380 nm and an emission wavelength of 505 nm. A minimum of 10 goblet cells was selected and the Ca^2+^ response was followed for approximately 2 min. MaR2 was either added alone or treated with inhibitors before addition of either MaR2 or the positive controls MaR1, carbachol, or histamine. 

The inhibitors H89, BOC2, BLT1, RO 317549, 2APB, U73122, U73343 were added 30 min prior to the agonist. 1-butanol, t-butanol and thapsigargin were added 15 min prior to the agonist. Change in peak [Ca^2+^]_i_ was calculated by subtracting the average basal [Ca^2+^]_i_ from the peak [Ca^2+^]_i_.

### 4.5. Measurement of High Molecular Weight Glycoconjugate Secretion

Cultured rat conjunctival goblet cells were trypsinized and transferred to 24 well plates. The cells were serum starved in free RPMI 1640 media containing 0.5% bovine serum album (BSA) for 120 min. MaR2 (10^−10^–10^−8^ M) was then added alone or the cells were incubated with an inhibitor for 30 min, and then stimulated with MaR2 (10^−10^–10^−8^ M) or carbachol (10^−4^ M) for 2 h. The amount of goblet cell high molecular weight glycoconjugate secretion was measured using the lectin UEA-1 in an enzyme linked lectin assay (ELLA). Glycoconjugate secretion is shown as -fold increase above basal (which was set to 1).

### 4.6. Statistical Analysis

Data are expressed as mean ± SEM. N indicates cells cultured from different animals. Data were analyzed by either Student’s *t*-test or one-way ANOVA followed by Tukey test. *p* < 0.05 was considered significant. Statistical analyses were performed using Excel (version 16.16.27, Microsoft Corp) and GraphPad Prism (version 9.3.1).

## 5. Conclusions

As MaR2 and MaR1 each modulate conjunctival goblet cell function, they each have potential as novel, but differing, options for treatment of ocular surface inflammatory diseases including allergic conjunctivitis and dry eye disease.

## Data Availability

The data that support the findings of this study are available from the corresponding author upon reasonable request.

## Supplementary Materials

The following are available online at https://www.mdpi.com/article/10.3390/ijms23116233/s1.
