# Supplementary material for: Signaling Pathways Used by the Specialized Pro-Resolving Mediator Maresin 2 Regulate Goblet Cell Function: Comparison with Maresin 1
Source: Int J Mol Sci. 2022 Jun 2;23(11):6233. doi: 10.3390/ijms23116233 (PMC9181304; doi:10.3390/ijms23116233)
Supplement: Supplementary file 1 [file ijms-23-06233-s001.zip › ijms-1728252-supplementary.pdf]

Supplementary Materials

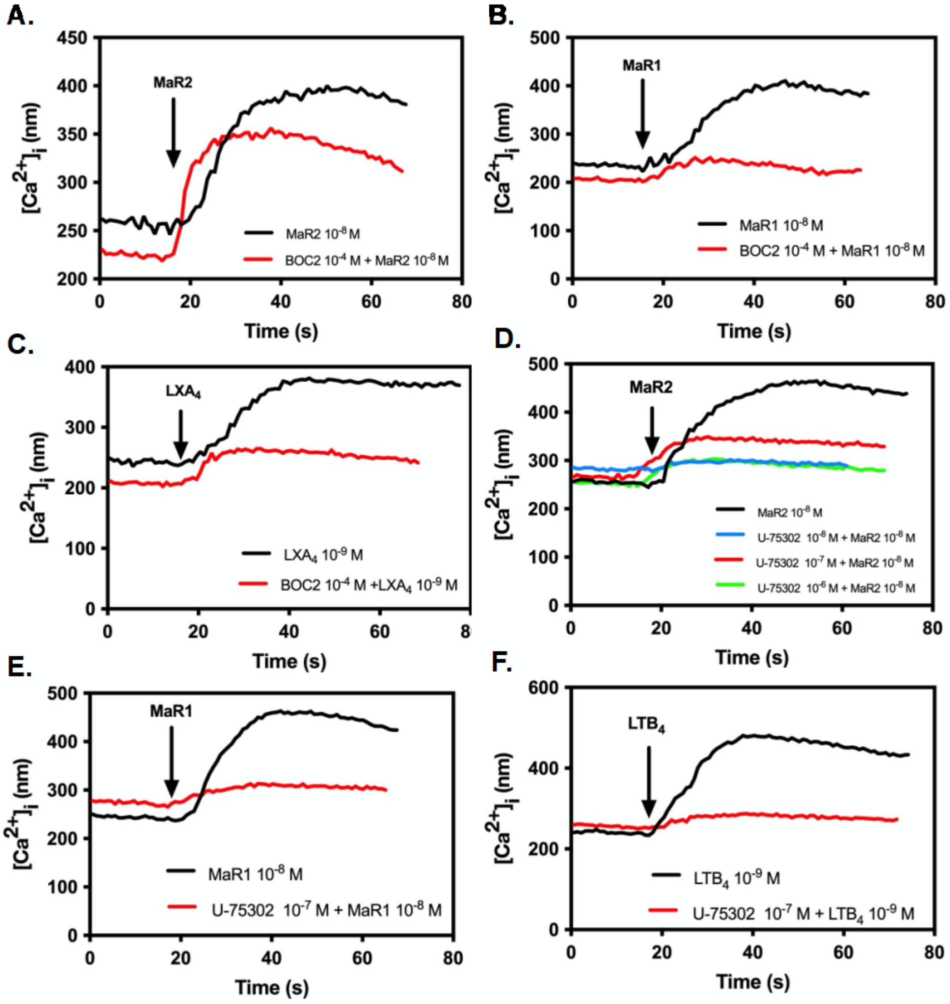

Figure S1: Inhibition of the ALX/FPR2- and the BLT1 receptors acts on stimulation of [Ca^{2+}]$_i$ by MaR2 at 10^{-8} M. A, **B**, and **C** show changes in [Ca^{2+}]$_i$ with time treated with the ALX/FPR2 receptor inhibitor BOC2 (10^{-4} M) and then stimulated with MaR2 10^{-8} M, MaR1 10^{-8} M, and LXA_4 10^{-9} M. **D**, **E** and **F** show changes in [Ca^{2+}]$_i$ with time treated with the BLT1 receptor inhibitor U-75302 (10^{-8} – 10^{-6} M) and then stimulated with MaR2 10^{-8} M, MaR1 10^{-8} M, and LTB_4 10^{-8} M. Data are mean ± SEM of five (**A**, **B**, and **C**) and six (**D**, **E**, and **F**) experiments.

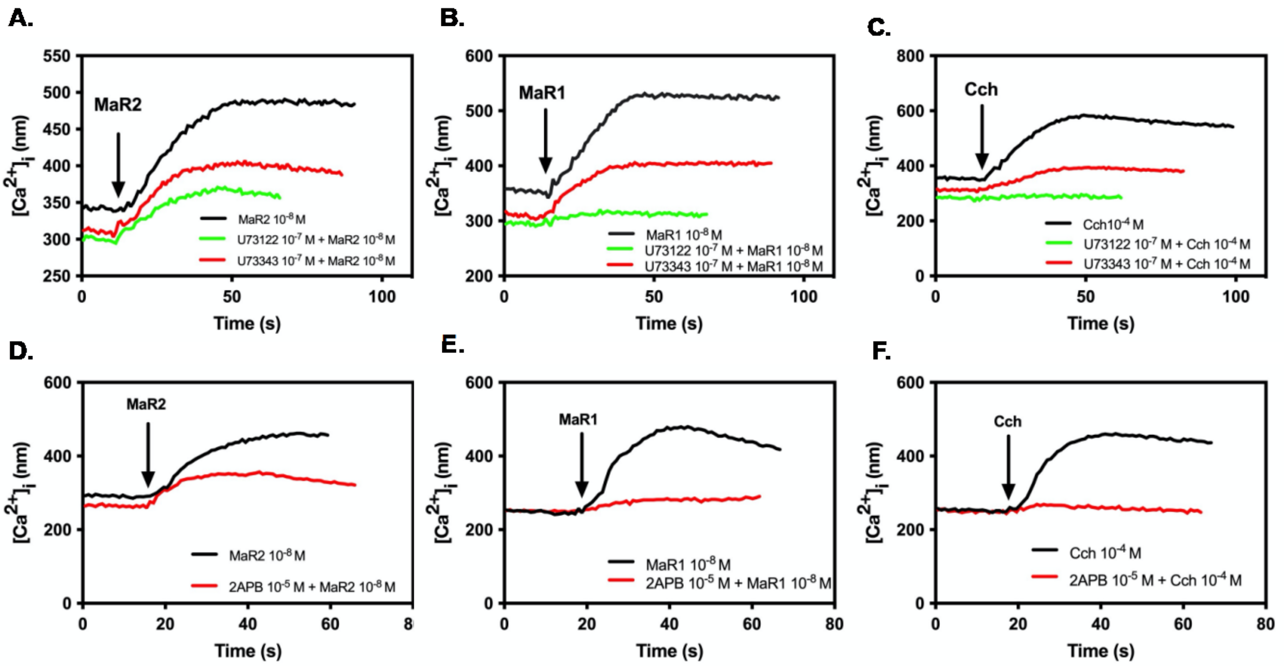

Figure S2: Maresin 2 increase in [Ca^{2+}]$_i$ is independent of the PLC-pathway in rat conjunctival goblet cells. Changes in [Ca^{2+}]$_i$ with time are shown. In **A**, **B** and **C**, goblet cells were treated with vehicle, the active PLC inhibitor U-73122 or the inactive PLC inhibitor U-73343 both at 10^{-7} M for 30 minutes and stimulated with MaR2 (10^{-8} M), MaR1 (10^{-8} M) or Cch (10^{-4} M). In **D**, **E** and **F,** goblet cells were treated with vehicle or 2APB (10^{-5} M) and stimulated with MaR2 (10^{-8} M), MaR1 (10^{-8} M) or Cch (10^{-4} M). Data are mean ± SEM of four (**A**, **B**, and **C**) and five (**D**, **E**, and **F**) experiments.

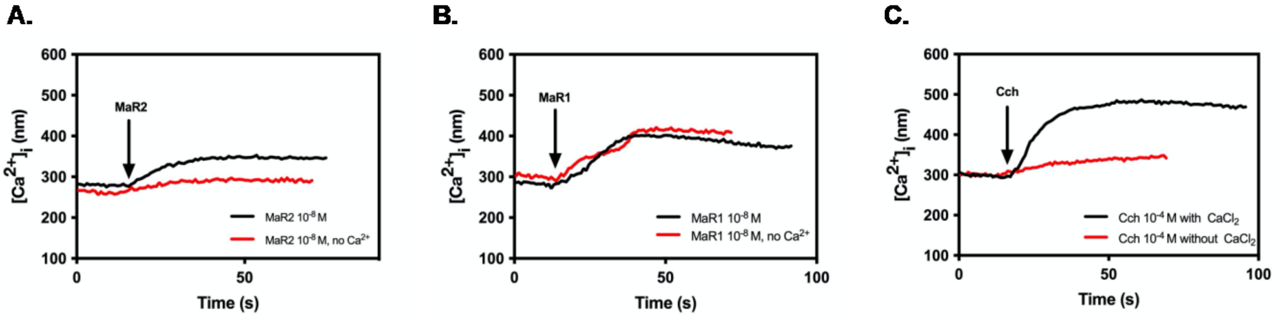

Figure S3: Maresin 2 stimulated increase in [Ca^{2+}]$_i$ is independent of extracellular Ca^{2+} in rat conjunctival goblet cells. Changes in [Ca^{2+}]$_i$ with time are shown in goblet cells incubated with KRB with or without $CaCl_2$ and then stimulated with (**A**) MaR2 (10^{-8} M), (**B**) MaR1 (10^{-8} M) or (**C**) Cch (10^{-4} M). Data are mean ± SEM of four experiments in each panel**.**

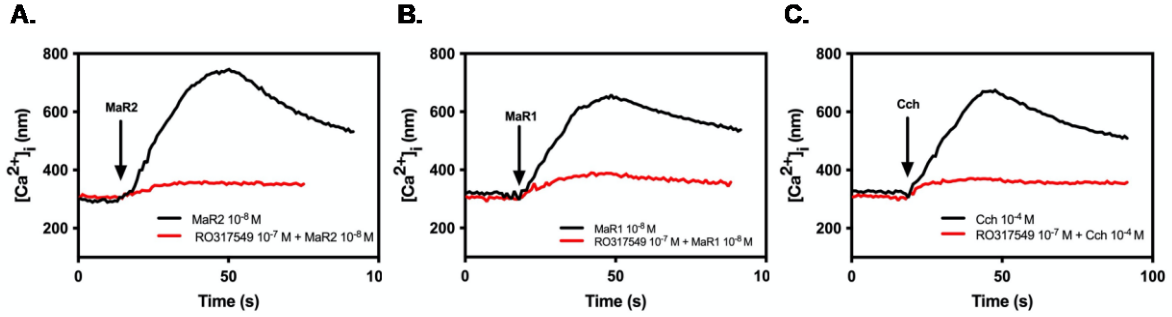

Figure S4: Maresin 2 increases [Ca^{2+}]$_i$ by activation of protein kinase C (PKC). Changes in [Ca^{2+}]$_i$ with time are shown in goblet cells incubated with RO317549 (10^{-7} M) for 30 minutes and then stimulated with (**A**) MaR2 (10^{-8} M), (**B**) MaR1 (10^{-8} M) or (**C**) Cch (10^{-4} M). Data are mean ± SEM of three experiments in each panel.

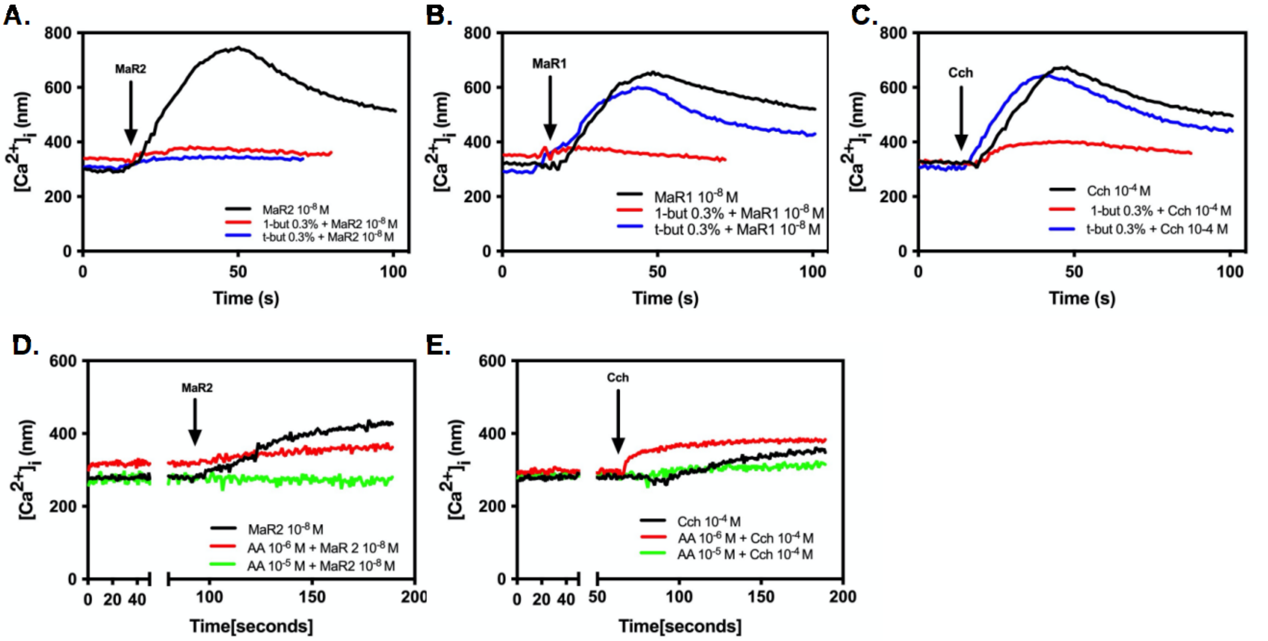

Figure S5: Maresin 2 has different dependency than MaR1 and Cch on Phospholipase D (PLD) and Phospholipase A_2 (PLA_2) to increase $[Ca^{2+}]_i$ and stimulate secretion in rat conjunctival goblet cells. Changes in $[Ca^{2+}]_i$ with time are shown. Goblet cells were preincubated with the PLD inhibitor 0.3% 1-butanol or the inactive analog 0.3% t-butanol for 15 minutes and then stimulated with (**A**) MaR2 (10^{-8} M), (**B**) MaR1 (10^{-8} M) or (**C**) Cch (10^{-4} M). Goblet cells were preincubated with the PLA_2 inhibitor aristolochic acid 10^{-5} M or 10^{-6} M for 30 minutes and stimulated with (**D**) MaR2 (10^{-8} M) or (**E**) Cch (10^{-4} M). Data are mean ± SEM of three (**A**, **B,** and **C**) and four (**D** and **E**) experiments.

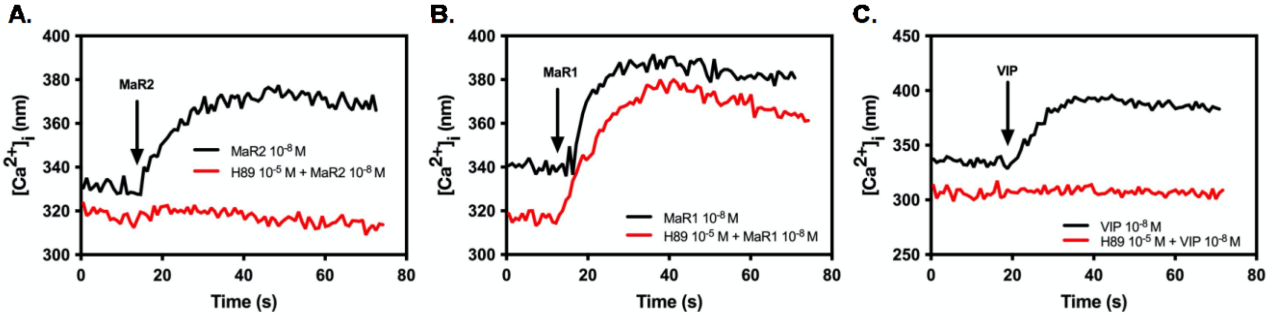

Figure S6: Maresin 2 uses protein kinase A to increase $[Ca^{2+}]_i$ and stimulate secretion in rat conjunctival goblet cells. Goblet cells were incubated with the protein kinase A (PKA) inhibitor H89 (10^{-5} M) for 30 minutes and then stimulated with (**A**) MaR2 (10^{-8} M), (**B**) MaR1 (10^{-8} M) or (**C**) VIP (10^{-8} M) to measure $[Ca^{2+}]_i$ over time. Data are mean ± SEM of five experiments in each panel.

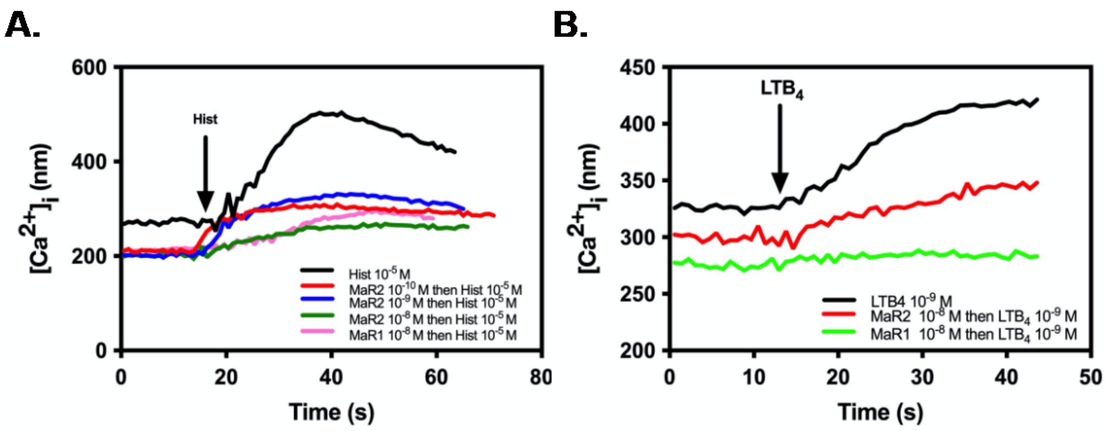

Figure S7: Maresin 2 inhibits histamine-, but not LTB_4-stimulated increase in $[Ca^{2+}]_i$ and histamine-stimulated glycoconjugate secretion in rat conjunctival goblet cells. Changes in $[Ca^{2+}]_i$ with time are shown. (**A**) Goblet cells were incubated with MaR2 (10^{-10} to 10^{-8} M) or MaR1 (10^{-8} M) for 30 minutes, then stimulated with histamine (10^{-5} M) and (**B**) goblet cells were incubated with MaR2 (10^{-8} M) or MaR1 (10^{-8} M) for 30 minutes, then stimulated with LTB_4 10^{-9} M. Data are mean ± SEM of three (**A**) and four (**B**) experiments.
